# Functional Connectivity of Heschl’s Gyrus Associated With Age-Related Hearing Loss: A Resting-State fMRI Study

**DOI:** 10.3389/fpsyg.2019.02485

**Published:** 2019-11-06

**Authors:** Megan C. Fitzhugh, Angela Hemesath, Sydney Y. Schaefer, Leslie C. Baxter, Corianne Rogalsky

**Affiliations:** ^1^College of Health Solutions, Arizona State University, Tempe, AZ, United States; ^2^School of Biological and Health Systems Engineering, Arizona State University, Tempe, AZ, United States; ^3^Department of Psychology, Mayo Clinic, Scottsdale, AZ, United States

**Keywords:** hearing loss, aging, Heschl’s gyrus, functional connectivity, resting-state fMRI

## Abstract

A large proportion of older adults experience hearing loss. Yet, the impact of hearing loss on the aging brain, particularly on large-scale brain networks that support cognition and language, is relatively unknown. We used resting-state functional magnetic resonance imaging (fMRI) to identify hearing loss-related changes in the functional connectivity of primary auditory cortex to determine if these changes are distinct from age and cognitive measures known to decline with age (e.g., working memory and processing speed). We assessed the functional connectivity of Heschl’s gyrus in 31 older adults (60–80 years) who expressed a range of hearing abilities from normal hearing to a moderate hearing loss. Our results revealed that both left and right Heschl’s gyri were significantly connected to regions within auditory, sensorimotor, and visual cortices, as well as to regions within the cingulo-opercular network known to support attention. Participant age, working memory, and processing speed did not significantly correlate with any connectivity measures once variance due to hearing loss was removed. However, hearing loss was associated with increased connectivity between right Heschl’s gyrus and the dorsal anterior cingulate in the cingulo-opercular network even once variance due to age, working memory, and processing speed was removed. This greater connectivity was not driven by high frequency hearing loss, but rather by hearing loss measured in the 0.5–2 kHz range, particularly in the left ear. We conclude that hearing loss-related differences in functional connectivity in older adults are distinct from other aging-related differences and provide insight into a possible neural mechanism of compensation for hearing loss in older adults.

## Introduction

Beginning at age 50, as many as 15% of adults in the United States have at least a mild hearing loss, with this rate doubling every decade, such that by the seventh decade approximately two-thirds of adults have a hearing loss (Lin et al., 2011b). These trends reflect hearing thresholds typically assessed between 0.5 and 4 kHz, wherein most of the frequencies of speech sounds fall. However, the prevalence of high-frequency hearing loss (thresholds between 4 and 8 kHz) is much greater at an earlier age, affecting one-third of adults 50–59 years and 59% of adults 60–69 years (Agrawal et al., 2008). These changes in hearing abilities in older adults negatively impact communication, resulting in speech comprehension difficulties, particularly in noisy environments (Tun et al., 2010; Desjardins and Doherty, 2013; Moore et al., 2014). Hearing loss is further linked to declines in self-reported activities of daily living and overall quality of life (Dalton et al., 2003; Ciorba et al., 2012; Hawkins et al., 2012). While hearing aids are the most common treatment for managing age-related hearing loss, only about 40% of adults over 70 years old with a hearing loss use hearing aids, citing high costs, and perceived lack of need as barriers (Gopinath et al., 2011; Lin et al., 2011b). Thus, given the high prevalence of adults with age-related hearing loss, many of whom do not seek treatment, there is a rapidly expanding population of older adults living with the effects of hearing loss and it is critical that we thoroughly characterize the effects of hearing loss in the aging brain to identify potential areas of intervention.

Recent work has linked age-related hearing loss to a roughly two-fold increased risk for cognitive decline and dementia, with hearing loss likely preceding the onset of cognitive decline by 5–10 years (Albers et al., 2015; Loughrey et al., 2017). In a large meta-analysis of the association between age-related hearing loss and cognitive abilities by Loughrey et al. (2017), the authors report small but significant declines in cognition with hearing loss across several cognitive domains, including executive function, memory, processing speed, and visuospatial abilities. Although studies of cognitive aging similarly report age-related changes in these domains, with the greatest declines observed in memory and processing speed abilities (for a review, see Glisky, 2007). While the causal relationship between age-related hearing loss and declines in cognitive abilities are not well understood, two possible mechanisms have been proposed: (1) both conditions derive from a common etiology or (2) sensory deprivation (i.e., hearing loss) may lead to cognitive decline (Baltes and Lindenberger, 1997; Lin et al., 2011a). This growing body of literature characterizing the high prevalence of hearing loss and its link to cognitive decline points to a need to uncover how hearing loss impacts the brain separate from other effects of aging. Yet, many (if not most) neuroimaging studies examining structural and functional brain measures of cognition as a function of typical aging do not collect or use hearing measures as a covariate in their analyses.

Few studies have investigated how age-related hearing loss impacts the brain (for a review, see Cardin, 2016). Changes to brain structure related to age-related hearing loss are unclear: some studies report gray matter volume *decreases* associated with hearing loss in primary auditory cortex (i.e., Heschl’s gyrus) and other temporal lobe structures (Husain et al., 2011; Peelle et al., 2011; Eckert et al., 2012), while others report volume *increases* within Heschl’s gyrus and the superior and middle temporal gyri (Boyen et al., 2013). Across the entire brain, a longitudinal study of older adults that compared gray matter volume reported no volume differences at baseline between the group with hearing loss compared to the group with normal hearing, but at the follow up 6 years later, those with a hearing loss exhibited accelerated volume declines, particularly within the right temporal lobe (Lin et al., 2014). In comparison, there is a much larger body of literature investigating age-related changes to the brain without considering any effects of hearing loss. These studies report gray matter volume and thickness decreases prominently within prefrontal cortical regions, with temporal cortex expressing more moderate declines (Walhovd et al., 2005; Alexander et al., 2006; Raz and Rodrigue, 2006; Fjell et al., 2009); however, these studies do not take hearing loss into account.

Functional MRI (fMRI) has identified differences between older adults with and without hearing loss in response to auditory stimuli. In a study utilizing tones, there was reduced activation in subcortical auditory regions with increasing hearing loss but no relationship with activation in auditory cortex (i.e., bilateral Heschl’s gyri and superior temporal gyri) (Boyen et al., 2014). Conversely, fMRI activation to more complex auditory stimuli, such as sentences, is reduced within Heschl’s gyrus in adults with poor hearing, even after controlling for age (Peelle et al., 2011). However, other studies report no difference in fMRI activation within Heschl’s gyrus and superior temporal regions in response to tones or words with age-related hearing loss (Harris et al., 2009; Profant et al., 2015). Hearing-related differences in temporal regions have also been identified using high-density electroencephalography (EEG) and cortical auditory evoked potentials: adults with hearing loss show increased amplitude and latency in the P2 peak, which is postulated to originate from Heschl’s gyrus (Campbell and Sharma, 2013; Lightfoot, 2016). Nevertheless, it is unclear how the changes in structural and functional brain measures associated with age-related hearing loss relate to well-studied changes associated with age alone.

Resting-state fMRI can be used to disentangle general age effects from the effects of age-related hearing loss on brain function. This technique measures functional connectivity of brain networks in the absence of any task, avoiding introducing task- or stimuli-related confounds, yet still capturing the brain networks that are modulated during language and cognitive tasks (Calhoun et al., 2008; Smith et al., 2009; Tomasi and Volkow, 2012b). Studies of age-related functional connectivity changes report that older adults have less distinct brain networks, as reflected in decreased within network connectivity and increased between network connectivity (Tomasi and Volkow, 2012a; Geerligs et al., 2014, 2015). Many studies report age-related declines in resting-state fMRI activity predominately within the default mode network (Andrews-Hanna et al., 2007; Damoiseaux et al., 2008; Tomasi and Volkow, 2012a; Campbell et al., 2013; Vidal-Piñeiro et al., 2014), as well as other networks including the motor, dorsal attention, and salience networks (Allen et al., 2011; Onoda et al., 2012; Tomasi and Volkow, 2012a). One study demonstrated that functional connectivity between the salience network and auditory networks declined with age (Onoda et al., 2012). However, none of these studies have accounted for participant hearing abilities. The few studies investigating hearing loss using resting-state fMRI report that the extent of an auditory network, defined by the functional connectivity from both left and right Heschl’s gyrus combined, did not differ between middle-aged normal and impaired hearing groups (Schmidt et al., 2013; Husain et al., 2014). Similarly, a recent study comparing functional connectivity from left Heschl’s gyrus between a group of hearing-impaired older adults and normal-hearing older adults report no group differences; the authors further report no differences in connectivity when hearing loss was entered into a regression analysis (Rosemann and Thiel, 2019). However, Rosemann and Thiel (2019) do report that decreases in functional connectivity between left Heschl’s gyrus and left inferior frontal gyrus significantly correlate with increased listening effort. There is also evidence to suggest that age-related hearing loss is linked to increased coupling of visual and auditory processing, based on a positive relationship between hearing loss and functional connectivity between right visual area MT+ and left Heschl’s gyrus (Puschmann and Thiel, 2017).

Heschl’s gyrus is a key target in investigating the effects of age-related hearing loss due to its role in auditory processing and the conflicting findings regarding the structural and functional impact of hearing. The structural and effective connectivity of Heschl’s gyrus to nearby temporal auditory processing regions has been explored in humans (Tardif and Clarke, 2001; Brugge et al., 2003; Upadhyay et al., 2008). However, the functional connectivity pattern from Heschl’s gyrus to other cortical regions is relatively unknown. One study determined that Heschl’s gyrus was functionally connected to the calcarine fissure of the visual cortex in younger adults (Eckert et al., 2008a). This mirrors anatomical tracer studies of nonhuman primates which reveal projections between auditory and visual cortices (Rockland and Ojima, 2003). Critically, it is unknown if Heschl’s gyrus is functionally connected to other brain networks involved in higher-order cognitive processing (e.g., salience or default mode networks) and how this connectivity pattern might differ in older adults as a function of age-related hearing loss. Therefore, the current study aims to characterize functional connectivity of Heschl’s gyrus in a group of older adults and to determine the impact of age-related hearing loss on functional connectivity.

In the present study, functional connectivity was characterized from left and right Heschl’s gyrus to all voxels in the cerebrum in 31 typical older adults (i.e., without dementia or mild cognitive impairment). Functional connectivity between each hemisphere’s Heschl’s gyrus and all cerebral voxels were computed and correlated with participant hearing ability, controlling for age, working memory, and processing speed, to determine if hearing loss is related to changes in brain functional connectivity that are distinct from other age-related changes. Given the findings of previous literature, we predicted that age-related hearing loss will be associated with increases in functional connectivity between Heschl’s gyrus and regions in the language, cingulo-opercular, and visual networks, after controlling for participant age and cognitive performance. A within-subjects approach was utilized instead of a younger versus older adult group comparison because our focus is on how age-related hearing loss in particular may be affecting functional connectivity results in typical aging. Age-related hearing loss typically has quite different onset trajectories, frequencies most affected, and neuroanatomical and structural mechanisms than hearing loss in younger adults (either developmental or acute) (Morzaria et al., 2004; Yamasoba et al., 2013). We also avoided comparing older adults with and without hearing loss, as age-related hearing loss occurs on a continuum, and the center of the continuum is where most older adults fall (i.e., mild-to-moderate hearing loss); thus to better capture how age-related hearing loss may be affecting the older adult population at large, we wanted to examine, within the same group of older adults, how age-related hearing loss and chronological age contribute to functional connectivity differences in older adults.

## Methods

### Participants

Participants were 31 adults aged 60–80 years [20 women; mean (sd) age = 67.8 (5.6) years, mean (sd) education = 17.5 (3.3) years] recruited from the greater Phoenix area. All participants were native English-speaking, right-handed as determined by the Edinburgh Handedness Inventory [mean (sd) = 94.6 (16.2)], and free from dementia, as determined by the Mini Mental Status Exam, score ≥ 27 [mean (sd) = 29.1 (1.0), range 27–30]. MMSE scores did not significantly correlate with participant age (*r* = −0.068, *p* = 0.717). No participant expressed indications of depression, as measured by the 15-item Geriatric Depression Scale where a score greater than five suggests depression [mean (sd) = 0.9 ± 1.0, range 0–5]. Written informed consent was obtained from each participant in accordance with Arizona State University’s Institutional Review Board and the US Federal Policy for the Protection of Human Subjects guidelines. Participants were compensated monetarily for their efforts.

Participants completed written and oral questionnaires to screen for the presence of hearing loss in childhood or as a young adult, any known congenital disease, acquired illness, injury, or other etiology that may have caused a hearing loss. In addition, each participant completed a T1 structural MRI scan (described in section “MRI Procedures” below) which was read by a clinical neuroradiologist to identify any possible neural abnormalities or etiologies that may affect the hearing or cognitive measures collected in the present study; all participants passed this MRI screening.

Pure-tone audiometry (PTA) was used to assess participant hearing ability in both ears and was conducted using a GSI 18 Audiometer and supra-aural headphones in a quiet, but not sound-attenuated, testing room. Assessors were trained by a clinical audiologist. First, the outer ear and ear canal were checked for excess cerumen using an otoscope. No participants presented with blocked canals. Testing then began in the right ear at 1 kHz at 50 dB HL using a pulsed tone. The intensity was decreased by 10 dB for every accurately perceived tone and increased by 5 dB for every missed tone, until the participant responded twice in the affirmative for a single intensity level; this value was recorded as the threshold for that frequency. The following frequencies were tested: 0.250, 0.500, 1, 2, 4, and 8 kHz. If the participant wore hearing aids, hearing acuity was evaluated unaided. MRI was also conducted unaided. Five participants reported wearing hearing aids.

Subtests of the *Wechsler Adult Intelligence Scale* 4th edition (*WAIS-IV*) were administered to collect measures of working memory and processing speed (Wechsler et al., 2008). The Digit Span (Forward, Backward, and Sequencing) and the Arithmetic subtests comprise the Working Memory Index (WMI), which was used as a measure of working memory. The Symbol Search and Coding subtests comprise the Processing Speed Index (PSI), which was used as a measure of speed of processing.

### MRI Procedures

MRI scanning was conducted at the Barrow Neurological Institute in Phoenix, AZ on a research-dedicated 3T Philips Ingenia scanner. During the resting-state fMRI acquisition, participants lay awake, in the absence of an explicit task, with eyes open and fixed on a crosshair. Resting-state fMRI data were collected using single-shot EPI with the following parameters: one 10-min run, 197 total volumes, TR = 3,000 ms, TE = 30 ms, flip angle = 90°, FOV = 217 × 217, matrix = 64 × 62, 48 axial slices, 3.39 mm slice thickness, in-plane resolution = 3.39 × 3.39 mm. Field maps were also collected to account for field inhomogeneity using the following parameters: TR = 20 ms, TE (short) = 2.3 ms, TE (long) = 4.6 ms, flip angle = 10°, FOV = 240 × 240, matrix = 80 × 80, voxel size = 3 mm × 3 mm × 3 mm, 52 sagittal slices, acquisition time = 1 min 22 s. A T1 MPRAGE anatomical scan also was acquired for each participant with the following parameters: TR = 6.74 s, TE = 3.10 ms, flip angle = 9°, FOV = 270 × 253, matrix = 256 × 256, voxel size = 1.1 mm × 1.1 mm × 1.2 mm, 170 sagittal slices, acquisition time = 5 min 34 s.

Field maps were created using SPM12’s FieldMap toolbox and applied to CONN’s pre-processing pipeline to correct for field inhomogeneities within the resting-state functional images. The following pre-processing procedures were conducted in the Functional Connectivity Toolbox (CONN; Whitfield-Gabrieli and Nieto-Castanon, 2012) using SPM12 functions: removal of the first three volumes, field map correction, slice-timing correction, realignment to the first functional volume, coregistration to each participant’s anatomical T1 scan, normalization to the MNI template, resampling at 2 mm × 2 mm × 2 mm, and spatial smoothing with an 8 mm FWHM Gaussian kernel. Denoising was performed to remove potentially confounding effects including motion and physiological noise which may induce spurious correlations between voxels (Fox et al., 2005; Van Dijk et al., 2010). The artifact detection tool (ART, incorporated into the CONN toolbox, http://www.nitrc.org/projects/artifact_detect) was used to compute a composite motion measure. This composite motion measure was used to identify and scrub volumes with motion greater than 2 mm or with changes in the global signal mean greater than a *Z*-value of 9 (these settings reflect the liberal default settings within ART, which remove approximately 1% of the data). These volumes were modeled out at the individual participant level using nuisance regressors (including five or fewer volumes for five participants, 15 volumes for one participant, and 54 volumes for one participant, out of 194 total volumes; the participant with 54 scrubbed volumes was not identified as an outlier in the results reported below). The CONN toolbox utilizes an aCompCor (anatomical component analysis correction) regression to reduce physiological noise associated with cerebrospinal fluid and white matter by extracting five principle components from each tissue type (Behzadi et al., 2007). Whole-brain BOLD signal regression was not performed to avoid inducing anti-correlations, to allow for investigations into negative correlations, and since the aCompCor strategy has been shown to be more sensitive in detecting positive correlations than global signal regression (Chai et al., 2012; Murphy et al., 2013). Additional nuisance regressors include the six motion realignment parameters, the components identified in the aCompCor procedure, and a linear detrending term. Lastly, bandpass filtering was also applied (0.008–0.09 Hz). These procedures represent the standard preprocessing stream offered in the CONN toolbox.

### Functional Connectivity Analysis

Functional connectivity was computed between anatomically defined ROIs (regions of interest) of left and right Heschl’s gyrus and all voxels of the cortex. Pearson correlation coefficients were calculated between the average time course within each ROI and the time courses of all other cerebral voxels; correlation coefficients were then Fisher transformed (i.e., *Z*-values). Heschl’s gyri ROIs (left: center of mass = −46, −20, 8, 309 voxels; Right: center of mass = 46, −16, 8, 282 voxels) were provided in the CONN toolbox and are from the FSL Harvard-Oxford Atlas maximum likelihood cortical atlas. To characterize the average functional connectivity from left and right Heschl’s gyri across the cortex, ROI-to-voxel connectivity was assessed using two-sided, one-sample *t*-tests thresholded at voxelwise *p* < 0.001 uncorrected and cluster-level *p* < 0.05 FDR. To compare changes in connectivity associated with hearing loss to that of age and cognitive measures commonly affected by age (e.g., working memory and processing speed scores), the following two-sided correlations were conducted with a threshold of voxelwise *p* < 0.001 uncorrected and cluster-level *p* < 0.05 FDR: (1) the correlation of hearing ability with connectivity from left Heschl’s gyrus and from right Heschl’s gyrus to all voxels, controlling for age, working memory, and processing speed, and (2) the correlations of age, working memory, and processing speed with connectivity from left Heschl’s gyrus and right Heschl’s gyrus, respectively, to all voxels, controlling for hearing ability.

## Results

### Hearing and Cognitive Abilities

**Figure 1:**
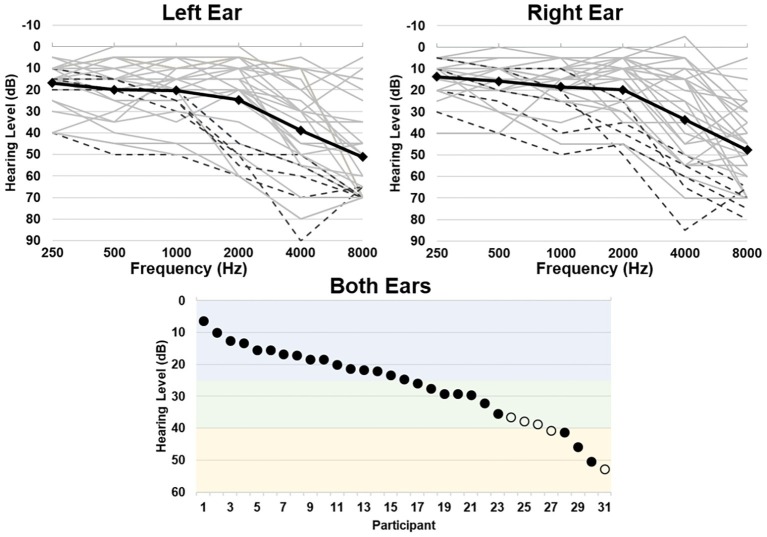
Hearing thresholds as assessed by pure-tone audiometry. Top left and right panels depict average thresholds for each ear, respectively. Individual participant thresholds are shown in light gray, individuals who wear hearing aids are shown in black dashed lines, and group average thresholds are shown in the black solid line. Bottom panel depicts the average threshold across all frequencies for both ears per participant, depicting a range of normal hearing levels (blue), mild (green), and moderate (yellow) hearing loss (Clark, 1981). Individuals who wear hearing aids are shown in open circles.

Hearing ability was quantified as the average threshold between all tested frequencies over both ears, where a higher threshold indicates greater hearing loss [mean (sd) = 26.76 (12.01) dB, range = 6.25–52.92 dB, corresponding to a range of hearing abilities from normal hearing to moderate hearing loss; Figure 1]. Mean (sd) standardized scores for the WMI were 105.5 (11.5), ranging from 83 to 128; scores for the PSI were 112.2 (11.8), ranging from 94 to 137. Hearing ability did not significantly correlate with participant age (*r* = 0.320, *p* = 0.080) or MMSE scores (*r* = −0.029, *p* = 0.875). Hearing ability also did not significantly correlate with WMI scores (*r* = −0.132, *p* = 0.480) or PSI scores (*r* = 0.221, *p* = 0.233).

### Heschl’s Gyrus Connectivity in Older Adults

**Figure 2:**
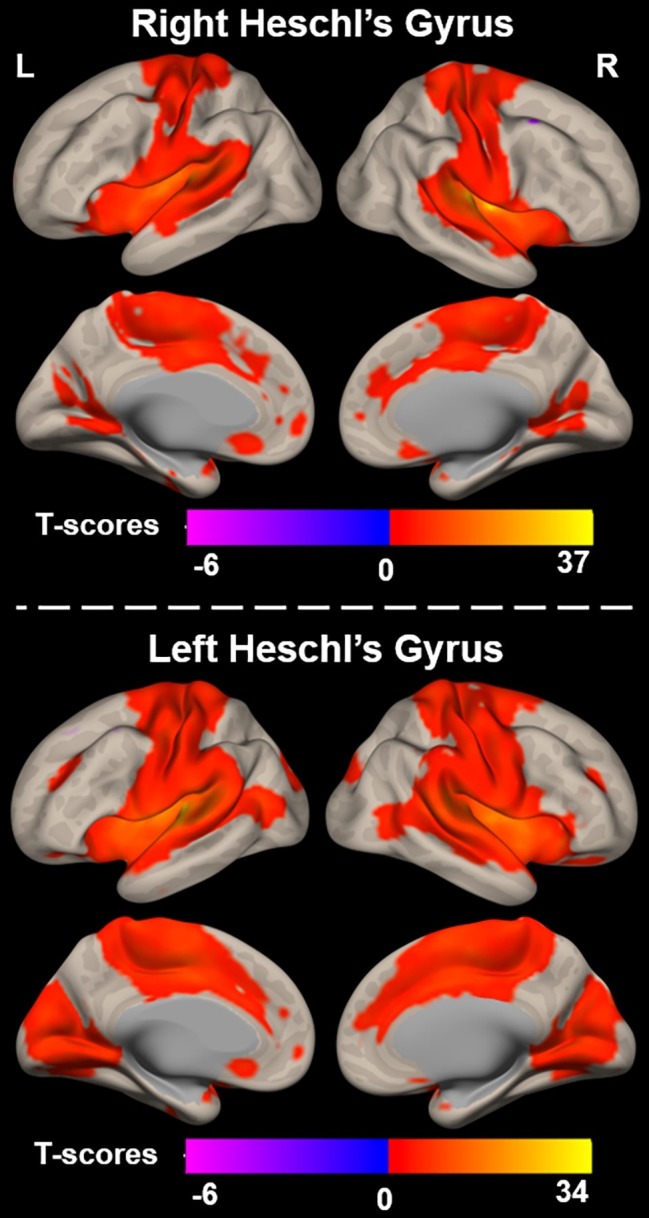
Functional connectivity between right (top) and left (bottom) Heschl’s gyrus and all voxels across the cerebrum, voxelwise *p* < 0.001 uncorrected, and cluster-level *p* < 0.05 FDR.

Functional connectivity (i.e., r-to-Z Fisher transformed correlation coefficients) from left and right Heschl’s gyrus to all cerebral voxels is characterized in Figure 2. Right Heschl’s gyrus was significantly and positively functionally connected to numerous regions known to be implicated in sensory, sensorimotor, and cognitive brain networks. The auditory regions include itself, left Heschl’s gyrus, and bilateral superior temporal regions extending the length of the superior temporal gyrus and extending into the inferior parietal lobes. Bilateral sensorimotor regions along the pre- and post-central gyri and visual cortex in the lingual and cuneal gyri also demonstrated significant functional connectivity with right Heschl’s gyrus. Right Heschl’s gyrus was also significantly connected to bilateral anterior cingulate cortex, supplementary motor area, and insula, which are known to constitute the well-studied cingulo-opercular network. Only a small region (peak voxel: 38, 14, 62, 149 voxels) within the right middle frontal gyrus expressed significant negative functional connectivity from right Heschl’s gyrus.

Similar to the right, left Heschl’s gyrus was significantly and positively functionally connected to bilateral Heschl’s gyri, as well as regions extending the length of the superior temporal gyri. It is notable though that the bilateral temporal regions significantly functionally connected to left Heschl’s gyrus extend more posteriorly and inferiorly into the bilateral middle temporal gyri than observed with right Heschl’s gyrus. Left Heschl’s gyrus was also significantly connected to bilateral pre-central and post-central gyri. Compared to right Heschl’s gyrus, functional connectivity from left Heschl’s gyrus covered a larger portion of visual areas and extended into medial and posterior occipital cortex, including lingual and lateral occipital gyri, respectively. Significant connectivity was also observed to regions of the cingulo-opercular network, including bilateral insula, anterior cingulate cortex, and supplementary motor areas. A region within the left superior and middle frontal gyri (peak voxel: −40, 14, 60, 211 voxels) exhibited negative functional connectivity to left Heschl’s gyrus.

### Heschl’s Gyrus Connectivity Differences as a Function of Age-Related Hearing Loss

**Figure 3:**
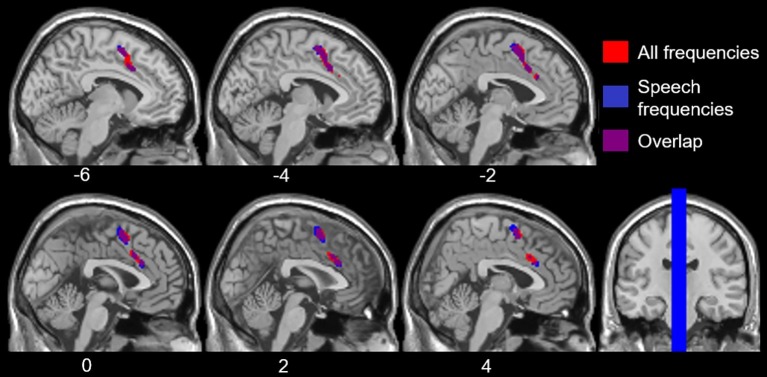
Voxels with functional connectivity to right Heschl’s gyrus that are significant and positively correlated with average hearing thresholds across all frequencies (red) and within speech frequencies (0.5–2 kHz, blue), controlling for age, WMI, and PSI voxelwise *p* < 0.001 uncorrected, cluster-level *p* < 0.05 FDR.

**Figure 4:**
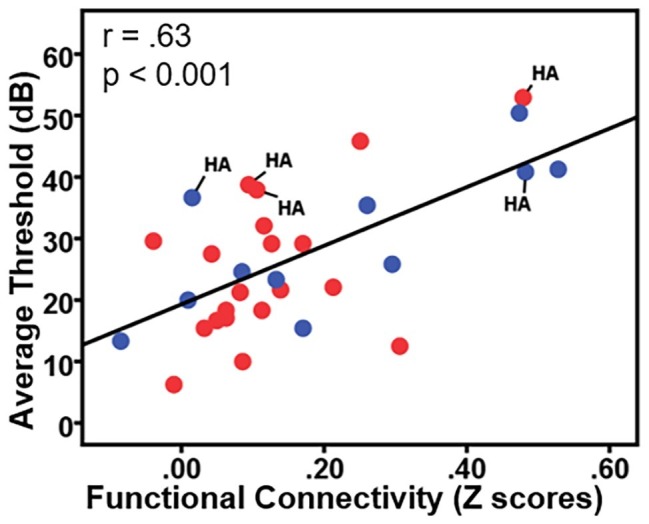
Plot of the correlation between participant hearing ability (i.e., the average threshold 0.250–0.8 kHz in dB from both ears) and the functional connectivity between right Heschl’s gyrus and the dorsal anterior cingulate. Red circles = females; blue circles = males; HA = hearing aid users.

To examine the effects of age-related hearing loss on Heschl’s gyrus connectivity compared to age and cognitive measures, functional connectivity between Heschl’s gyrus and all cerebral voxels was correlated with hearing loss. The analysis correlating hearing loss with connectivity, controlling for age, WMI, and PSI is shown in Figure 3. There were no significant correlations between functional connectivity of left Heschl’s gyrus and any cerebral voxels with hearing loss. However, from right Heschl’s gyrus, hearing loss was correlated with increased connectivity to a group of voxels in the dorsal anterior cingulate cortex, paracingulate gyrus, and supplementary motor area (peak = −4, 16, 34, 388 voxels; Figure 3). Average functional connectivity within this dorsal anterior cingulate region correlated with hearing ability is shown in Figure 4. It is noteworthy that this finding is not driven by the inclusion of individuals with hearing aids: with the five such participants excluded, a similar and highly overlapping dorsomedial cluster was identified (peak = −2, 8, 56, 156 voxels), and no other significant voxels were identified. None of the analyses correlating functional connectivity of left or right Heschl’s gyri with age, WMI, or PSI, while controlling for hearing loss revealed significant voxels that survived multiple comparison correction.

To explore the contributions of high-frequency hearing loss and hearing loss within the speech frequency range to the results reported above, we ran additional analyses correlating hearing loss with functional connectivity of left and right Heschl’s gyrus, controlling for age, WMI, and PSI. The difference from the previous analyses is that the measure of hearing loss was (1) the PTA thresholds only averaged across the high frequencies tested (4 and 8 kHz), and then (2) with the measure of hearing loss being the PTA thresholds only averaged across the speech frequencies tested (0.5–2 kHz). These frequency ranges were selected based on previous epidemiological work in older adults (Lin et al., 2011b) and American Speech-Language-Hearing Association recommendations (Clark, 1981). No voxels were identified as exhibiting functional connectivity with either left or right Heschl’s gyrus that were significantly correlated with high-frequency hearing loss, and this lack of effect remained when participants who wore hearing aids were excluded from the analysis.

However, hearing loss within the speech frequency range was significantly correlated with functional connectivity between right Heschl’s gyrus and a group of voxels in the dorsal anterior cingulate and supplemental motor area (peak = −2, 4, 58, 484 voxels; Figure 3), which is highly overlapping with the group of voxels identified by the analysis using the average PTA thresholds across all frequencies sampled (i.e., 0.250–8 kHz). When excluding participants who wore hearing aids, no two-sided correlations between hearing loss within the speech frequencies and functional connectivity of right Heschl’s gyrus were significant, although a (more liberal) one-side test identified a positive correlation between right Heschl’s gyrus and voxels largely within the left and right supplementary motor area and left paracingulate cortex (peak = −4, 6, 56, 236 voxels), which is highly overlapping with the finding in all participants across the speech frequencies. There were no significant findings (one- or two-sided) for the relationship between left Heschl’s gyrus functional connectivity and hearing loss in the speech frequencies across all participants, or when participants who wore hearing aids were excluded.

Lastly, given the well-documented right ear advantage for hearing sensitivity for speech (for reviews, see Hiscock and Kinsbourne, 2011; Hugdahl and Westerhausen, 2016), we further characterized the effects of age-related hearing loss on voxelwise functional connectivity from left and right Heschl’s gyrus by using the average thresholds from each ear in separate analyses. Findings of average threshold in the left ear, but not the right ear, mirrored the results when averaging across both ears: hearing loss from the left ear only was correlated with significantly increased functional connectivity from right Heschl’s gyrus to voxels within in the dorsal anterior cingulate cortex, paracingulate gyrus, and supplementary motor area (peak = 0, 8, 56, 710 voxels), similar to the region identified with hearing loss over both ears. Functional connectivity from left Heschl’s gyrus was not correlated with hearing loss in either the left or right ear. We also examined high frequency and speech frequency hearing loss in each ear separately: again, the left ear appears to be driving the results of the analysis reported above using an average of both ears: speech frequency hearing loss in the left ear, but not the right, was significantly and positively correlated with functional connectivity between right Heschl’s gyrus and voxels within the dorsal anterior cingulate, paracingulate, and supplementary motor regions (peak = −2, 6, 58, 751 voxels). High frequency hearing loss in either ear separately was not significantly correlated with functional connectivity in left or right Heschl’s gyrus. All of these results using each ear’s hearing thresholds separately remain when hearing aid users are excluded.

## Discussion

The aim of the present study was to characterize the functional connectivity of Heschl’s gyrus in older adults and to characterize how this connectivity varies with age-related hearing loss. Our findings demonstrate that both Heschl’s gyri are significantly functionally connected to regions within intrinsic sensory and cognitive networks in older adults. There were no differences in connectivity related to participant age, working memory, or processing speed performance, once hearing loss was controlled for. However, age-related hearing loss was positively associated with connectivity from right Heschl’s gyrus to the dorsal anterior cingulate, a main region within the cingulo-opercular network which is known to support attention. This association between hearing loss and greater functional connectivity of right Heschl’s gyrus was driven by hearing loss within the speech frequency range (i.e., not by high frequency hearing loss) and by hearing loss in the left (but not right) ear. These findings characterize the extensive functional connectivity of Heschl’s gyrus in older adults and point to a possible difference regarding its connectivity with the cingulo-opercular network that may serve as a compensatory mechanism in response to age-related hearing loss.

### Heschl’s Gyrus Functional Connectivity in Older Adults

Our findings illustrate that bilateral Heschl’s gyrus is highly functionally connected to primary somatosensory and motor cortices, aligning with prominent models of auditory speech processing and auditory-motor integration (Wilson et al., 2004; Hickok and Poeppel, 2007; Rauschecker and Scott, 2009). We also report that bilateral Heschl’s gyrus, but particularly the left auditory cortex, is functionally connected to primary and association visual cortex. Together, these results indicate that Heschl’s gyrus is highly functionally connected to other primary cortices (e.g., sensorimotor and visual) in older adults. These sensory and sensorimotor functional connections may underlie the ability to integrate crossmodal visual information (e.g., articulatory movements and facial expressions) with the auditory speech signal from a speaker, which is known to greatly improve comprehension of auditory signals (Summerfield, 1992; Eckert et al., 2008a).

Our findings also reveal that both Heschl’s gyri are significantly functionally connected to regions within the cingulo-opercular network. Functional connectivity of Heschl’s gyrus to regions within the cingulo-opercular network may offer insight into a mechanism that supports auditory attention processes. Previous work suggests that the cingulo-opercular network is involved in goal-driven behaviors through the initiation and top-down maintenance of task sets, adjusting performance in response to errors to optimize performance (Dosenbach et al., 2006, 2008). The anterior cingulate cortex (ACC) region of this network has been shown to reliably activate following stimuli or response conflict (i.e., as seen in the widely-used color-word Stroop task) in response to both auditory-only and visual-only conflict, but not audiovisual conflict (Botvinick et al., 2001; Milham et al., 2001; Roberts and Hall, 2008; Fitzhugh et al., 2019). Similarly, others propose that the ACC, as well as bilateral insula, are involved in top-down control of focal attention (i.e., target detection and awareness), overlapping with the “executive” attention network (Petersen and Posner, 2012), again suggesting at a possible mechanism for auditory attention processes.

### Hearing Loss Is Related to Greater Right Heschl’s Gyrus Functional Connectivity

**Figure 5:**
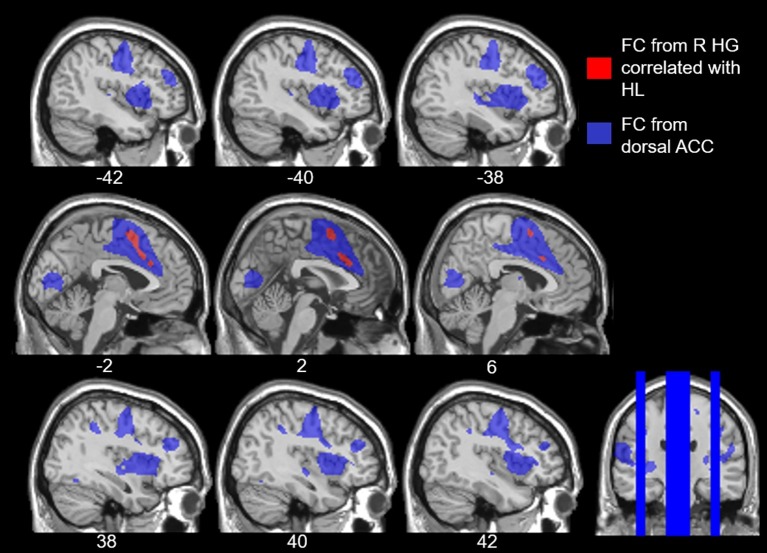
Overlay of the voxels that expressed increased functional connectivity (FC) to right Heschl’s gyrus (HG) with age-related hearing loss (HL) across all frequencies, voxelwise *p* < 0.001 uncorrected, cluster-level *p* < 0.05 FDR (red), and the cingulo-opercular network (blue). The cingulo-opercular network was defined using the results of the dorsal anterior cingulate (ACC) as a seed ROI in a seed-to-voxel functional connectivity analysis, voxelwise *p* < 0.001 FWE.

Our study revealed that age-related hearing loss was positively correlated with functional connectivity from right Heschl’s gyrus to the dorsal anterior cingulate. In other words, as the level of hearing loss increases so too does the functional connectivity between right auditory cortex and the dorsal anterior cingulate, a region within the cingulo-opercular network. To confirm that the dorsal anterior cingulate voxels that we identified as having increasing connectivity with right Heschl’s gyrus as a function of hearing loss are in fact part of the cingulo-opercular network, we used these dorsal anterior cingulate voxels as an ROI and then determined which voxels throughout the cerebrum exhibited significant functional connectivity to them. This exploration of our results indicated that these voxels are significantly functionally connected to the surrounding anterior cingulate and supplementary motor area cortex, as well as bilateral insula (Figure 5), which nicely aligns with the neuroanatomical regions repeatedly implicated in the cingulo-opercular network (Dosenbach et al., 2006, 2007; Seeley et al., 2007; Elton and Gao, 2014). This network map provides further evidence that age-related hearing loss is associated with increased connectivity between auditory cortex and the cingulo-opercular network. Critically, there were no differences in functional connectivity of either left or right Heschl’s gyrus associated with age, working memory, or processing speed, once controlling for hearing loss, suggesting that the findings reported here more likely reflect a relationship between functional connectivity and age-related hearing loss, and not other age-related declines. These findings are consistent with the idea that we have identified alterations in functional connectivity associated with age-related hearing loss, independent from other effects of age.

The small body of previous literature using resting-state fMRI to explore the impact of age-related hearing loss on functional connectivity is equivocal. A few studies report increased connectivity between Heschl’s gyrus and the visual MT+ area, as well as differences within the dorsal attention and default mode networks (Schmidt et al., 2013; Husain et al., 2014; Puschmann and Thiel, 2017). However, another very recent study found no group differences in functional connectivity from left Heschl’s gyrus between older adults with a hearing loss and older adults with normal hearing (Rosemann and Thiel, 2019). Differences in methodological approach may explain the discrepancies between these previous studies and the findings reported here. In the study by Husain et al. (2014), while they similarly used ROIs within left and right Heschl’s gyrus, their approach differed from ours in that they treated the Heschl’s gyri ROIs as a pair and averaged connectivity across the pair. Our study measured connectivity from left and right Heschl’s gyrus independently. In the study by Rosemann and Thiel (2019), the authors defined their “primary auditory” ROI based on a contrast of auditory-only sentences to baseline within an audio-visual sentence listening task (peak activation = MNI coordinates −56, −10, 2). This region falls outside of the anatomically based left Heschl’s gyrus ROI utilized in our study, and the right auditory cortex was not examined. There is evidence to suggest that different subdivisions of Heschl’s gyrus and adjacent auditory cortex are differentially impacted by hearing loss (Eckert et al., 2012; Cardin, 2016), thus it is likely that these differences in methodology allowed us to observe the effect that hearing loss has upon connectivity of right Heschl’s gyrus. In addition, Rosemann and Thiel’s measure of hearing loss was averaged across 2–8 kHz, which overlaps with our high frequency range (4–8 kHz) that did not yield any significant findings.

While differences in methodologies may explain the discrepancies between our findings and other studies using resting-state functional connectivity measures, our results align well with task-based fMRI findings of degraded speech comprehension (which may simulate hearing-loss effects in older adults). Several task-based fMRI studies demonstrate that activation within regions of the cingulo-opercular network is increased in response to acoustic degradation of speech stimuli in both young and older adults, when additional attentional resources are required to maximize speech comprehension performance (Eckert et al., 2008b; Adank, 2012; Wild et al., 2012; Erb et al., 2013; Vaden et al., 2015; Peelle, 2018). Additionally, in a task-based fMRI study of older adults, Erb and Obleser (2013) found that age-related hearing loss was associated with greater activation while listening to clear speech in the right anterior insula (part of the cingulo-opercular network), even after controlling for age. This suggests that older adults with a hearing loss likely recruit attentional cognitive resources supported by the cingulo-opercular network even in clear listening conditions, due to an increase in listening effort. Our results nicely complement these task-based fMRI findings. We suspect that the prolonged coupling of auditory and cingulo-opercular network activation that occurs throughout the gradual onset of age-related hearing loss due to greater attentional demands during listening may result in our observed greater functional connectivity between Heschl’s gyrus and dorsal anterior cingulate.

The resting-state and task-based fMRI studies that examined hearing loss mentioned above either averaged hearing measures across both ears or from the better ear. But examining each ear’s thresholds separately may provide more detail regarding how age-related hearing loss is related to functional brain changes. The well-known right ear advantage in hearing sensitivity and speech perception has been identified across the lifespan (for reviews, see Hiscock and Kinsbourne, 2011; Hugdahl and Westerhausen, 2016). This advantage has been found to increase in older adults, possibly as a function of age-related hearing loss; some attribute this increased right ear advantage instead to a left ear disadvantage, as exhibited by poorer dichotic speech recognition and other audiometric measures in the left ear (Jerger et al., 1994; Tadros et al., 2005; Roup et al., 2006; Westerhausen et al., 2015). Our correlation analyses using each ear’s thresholds separately indicate that the left ear’s hearing thresholds are driving the results of the analyses that use an average across both ears. Given the contralateral organization of primary auditory cortex, it is perhaps not surprising that we find that left ear hearing loss is correlated with right Heschl’s gyrus connectivity. But interestingly, perhaps it is the case that the previously reported left ear disadvantage in older adults is related to the greater coupling of right Heschl’s gyrus and dorsal ACC with left ear hearing loss, as reported in our study.

### Limitations and Future Directions

There are a few limitations to the present study that should be addressed in future work. While overall our older adult participants expressed a well-distributed range of hearing abilities (Figure 1), the levels of hearing ability across all tested frequencies only ranged from normal hearing to a moderate hearing loss. Future studies that include more individuals with moderate to severe levels of hearing loss, in both low and high frequencies, may be able to more fully characterize the impact of hearing loss on brain functional connectivity. Including middle-aged adults could also better capture the trajectory of functional connectivity differences associated with the onset and progression of age-related hearing loss and potential changes in working memory and processing speed. It is also the case that an overall increase in sample size would increase the power of the analyses to detect differences in functional connectivity as a function of age and age-related hearing loss. Nonetheless, the present study suggests that there is a need to further investigate how age-related hearing loss may be contributing to effects (or lack thereof) previously attributed to “typical aging” on functional connectivity of intrinsic and cognitive brain networks.

The present study, with nearly 2:1 ratio of females to males, is not well-equipped to robustly examine gender differences in resting-state functional connectivity. However, there are known gender differences regarding trajectory and frequency distributions of age-related hearing loss (Jerger et al., 1993; Demeester et al., 2009; Lin et al., 2011b). To our knowledge, only one study of resting-state fMRI attempted to examine gender differences in functional connectivity related to age-related hearing loss and found no gender effects. We have indicated in Figure 4 the data points from females (red) and males (blue) for transparency regarding how gender is related to our findings, but future studies are certainly needed to examine how gender differences affect age-related hearing loss and related functional brain changes.

The potential influences of hearing aid usage on functional connectivity should also be investigated. In this study, five individuals reported wearing hearing aids. As shown in Figure 4 (labeled with “HA”), the five participants who wear hearing aids exhibited the same trend of greater functional connectivity being associated with greater hearing loss seen across the other participants without hearing aids. Removing the five hearing aid users from each of our correlational analyses did not qualitatively change any of the findings. However, more thorough investigations are needed regarding how the use of hearing aids influences Heschl’s gyrus functional connectivity changes, particularly as related to age of onset of use, daily usage, and hearing aid effectiveness.

### Conclusion

In the present study, we use resting-state fMRI to investigate differences in the functional connectivity of Heschl’s gyrus as a function of age-related hearing loss in older adults without dementia. The older adults exhibited significant positive functional connectivity between Heschl’s gyri and large regions overlapping with the cingulo-opercular network, as well as with auditory, visual, somatosensory, and motor regions. After controlling for age, working memory, and processing speed, hearing loss (particularly within the frequencies of speech and in the left ear) was associated with increased functional connectivity between right Heschl’s gyrus and dorsal anterior cingulate cortex in the cingulo-opercular network. Conversely, age, working memory, and processing speed were not significantly correlated with functional connectivity of Heschl’s gyri, once controlling for hearing ability. Together our findings reveal age-related hearing loss differences in Heschl’s gyrus functional connectivity that may reflect compensatory attention-related mechanisms for auditory processing. Future studies are certainly needed to fully characterize the effects of age-related hearing loss versus other age-related factors on functional connectivity in older adults, but the present study provides clear evidence that studies of functional connectivity in older adults should consider how age-related hearing loss may be contributing to their results.

## Data Availability Statement

The raw data supporting the conclusions of this manuscript will be made available by the authors, without undue reservation, to any qualified researcher.

## Ethics Statement

Written informed consent was obtained from each participant in accordance with Arizona State University’s Institutional Review Board and the US Federal Policy for the Protection of Human Subjects guidelines. Participants were compensated monetarily for their efforts.

## Author Contributions

MF, SS, LB, and CR contributed to the design of the study. MF and AH were critical in implementing and analyzing the data. All authors contributed to data interpretation and manuscript preparation.

### Conflict of Interest

The authors declare that the research was conducted in the absence of any commercial or financial relationships that could be construed as a potential conflict of interest.

## References

[ref1] Adank P. (2012). The neural bases of difficult speech comprehension and speech production: two activation likelihood estimation (ALE) meta-analyses. Brain Lang. 122, 42–54. 10.1016/j.bandl.2012.04.014, PMID: 22633697

[ref2] Agrawal Y. Platz E. A. Niparko J. K. (2008). Prevalence of hearing loss and differences by demographic characteristics among US adults: data from the National Health and Nutrition Examination survey, 1999–2004. Arch. Intern. Med. 168, 1522–1530. 10.1001/archinte.168.14.1522, PMID: 18663164

[ref3] Albers M. W. Gilmore G. C. Kaye J. Murphy C. Wingfield A. Bennett D. A.. (2015). At the interface of sensory and motor dysfunctions and Alzheimer’s disease. Alzheimers Dement. 11, 70–98. 10.1016/j.jalz.2014.04.514, PMID: 25022540 PMC4287457

[ref4] Alexander G. E. Chen K. Merkley T. L. Reiman E. M. Caselli R. J. Aschenbrenner M.. (2006). Regional network of magnetic resonance imaging gray matter volume in healthy aging. Neuroreport 17, 951–956. 10.1097/01.wnr.0000220135.16844.b6, PMID: 16791083

[ref5] Allen E. A. Erhardt E. B. Damaraju E. Gruner W. Segall J. M. Silva R. F.. (2011). A baseline for the multivariate comparison of resting-state networks. Front. Syst. Neurosci. 5:2. 10.3389/fnsys.2011.00002, PMID: 21442040 PMC3051178

[ref6] Andrews-Hanna J. R. Snyder A. Z. Vincent J. L. Lustig C. Head D. Raichle M. E.. (2007). Disruption of large-scale brain systems in advanced aging. Neuron 56, 924–935. 10.1016/j.neuron.2007.10.038, PMID: 18054866 PMC2709284

[ref7] Baltes P. B. Lindenberger U. (1997). Emergence of a powerful connection between sensory and cognitive functions across the adult life span: a new window to the study of cognitive aging? Psychol. Aging 12, 12–21. 10.1037/0882-7974.12.1.12, PMID: 9100264

[ref8] Behzadi Y. Restom K. Liau J. Liu T. T. (2007). A component based noise correction method (CompCor) for BOLD and perfusion based fMRI. NeuroImage 37, 90–101. 10.1016/j.neuroimage.2007.04.042, PMID: 17560126 PMC2214855

[ref9] Botvinick M. M. Braver T. S. Barch D. M. Carter C. S. Cohen J. D. (2001). Conflict monitoring and cognitive control. Psychol. Rev. 108, 624–652. 10.1037/0033-295X.108.3.624, PMID: 11488380

[ref10] Boyen K. de Kleine E. van Dijk P. Langers D. R. M. (2014). Tinnitus-related dissociation between cortical and subcortical neural activity in humans with mild to moderate sensorineural hearing loss. Hear. Res. 312, 48–59. 10.1016/j.heares.2014.03.001, PMID: 24631963

[ref11] Boyen K. Langers D. R. M. de Kleine E. van Dijk P. (2013). Gray matter in the brain: differences associated with tinnitus and hearing loss. Hear. Res. 295, 67–78. 10.1016/j.heares.2012.02.010, PMID: 22446179

[ref12] Brugge J. F. Volkov I. O. Garell P. C. Reale R. A. Howard M. A. (2003). Functional connections between auditory cortex on Heschl’s gyrus and on the lateral superior temporal gyrus in humans. J. Neurophysiol. 90, 3750–3763. 10.1152/jn.00500.2003, PMID: 12968011

[ref13] Calhoun V. D. Kiehl K. A. Pearlson G. D. (2008). Modulation of temporally coherent brain networks estimated using ICA at rest and during cognitive tasks. Hum. Brain Mapp. 29, 828–838. 10.1002/hbm.20581, PMID: 18438867 PMC2649823

[ref14] Campbell K. Grigg O. Saverino C. Churchill N. Grady C. (2013). Age differences in the intrinsic functional connectivity of default network subsystems. Front. Aging Neurosci. 5:73. 10.3389/fnagi.2013.00073, PMID: 24294203 PMC3827623

[ref15] Campbell J. Sharma A. (2013). Compensatory changes in cortical resource allocation in adults with hearing loss. Front. Syst. Neurosci. 7:71. 10.3389/fnsys.2013.00071, PMID: 24478637 PMC3905471

[ref16] Cardin V. (2016). Effects of aging and adult-onset hearing loss on cortical auditory regions. Front. Neurosci. 10:199. 10.3389/fnins.2016.00199, PMID: 27242405 PMC4862970

[ref17] Chai X. J. Castañón A. N. Öngür D. Whitfield-Gabrieli S. (2012). Anticorrelations in resting state networks without global signal regression. NeuroImage 59, 1420–1428. 10.1016/j.neuroimage.2011.08.048, PMID: 21889994 PMC3230748

[ref18] Ciorba A. Bianchini C. Pelucchi S. Pastore A. (2012). The impact of hearing loss on the quality of life of elderly adults. Clin. Interv. Aging 7, 159–163. 10.2147/CIA.S26059, PMID: 22791988 PMC3393360

[ref19] Clark J. G. (1981). Uses and abuses of hearing loss classification. ASHA 23, 493–500. PMID: 7052898

[ref20] Dalton D. S. Cruickshanks K. J. Klein B. E. K. Klein R. Wiley T. L. Nondahl D. M. (2003). The impact of hearing loss on quality of life in older adults. Gerontologist 43, 661–668. 10.1093/geront/43.5.661, PMID: 14570962

[ref21] Damoiseaux J. S. Beckmann C. F. Arigita E. J. S. Barkhof F. Scheltens P. Stam C. J.. (2008). Reduced resting-state brain activity in the “default network” in normal aging. Cereb. Cortex 18, 1856–1864. 10.1093/cercor/bhm207, PMID: 18063564

[ref22] Demeester K. van Wieringen A. Hendrickx J. Topsakal V. Fransen E. van Laer L.. (2009). Audiometric shape and presbycusis. Int. J. Audiol. 48, 222–232. 10.1080/14992020802441799, PMID: 19363723

[ref23] Desjardins J. L. Doherty K. A. (2013). Age-related changes in listening effort for various types of masker noises. Ear Hear. 34, 261–272. 10.1097/AUD.0b013e31826d0ba4, PMID: 23095723

[ref24] Dosenbach N. U. F. Fair D. A. Cohen A. L. Schlaggar B. L. Petersen S. E. (2008). A dual-networks architecture of top-down control. Trends Cogn. Sci. 12, 99–105. 10.1016/j.tics.2008.01.001, PMID: 18262825 PMC3632449

[ref25] Dosenbach N. U. F. Fair D. A. Miezin F. M. Cohen A. L. Wenger K. K. Dosenbach R. A. T. (2007). Distinct brain networks for adaptive and stable task control in humans. PNAS 104, 11073–11078. 10.1073/pnas.0704320104 17576922 PMC1904171

[ref26] Dosenbach N. U. F. Visscher K. M. Palmer E. D. Miezin F. M. Wenger K. K. Kang H. C.. (2006). A core system for the implementation of task sets. Neuron 50, 799–812. 10.1016/j.neuron.2006.04.031, PMID: 16731517 PMC3621133

[ref27] Eckert M. A. Cute S. L. Vaden K. I. Kuchinsky S. E. Dubno J. R. (2012). Auditory cortex signs of age-related hearing loss. JARO 13, 703–713. 10.1007/s10162-012-0332-5, PMID: 22618352 PMC3441956

[ref28] Eckert M. A. Kamdar N. V. Chang C. E. Beckmann C. F. Greicius M. D. Menon V. (2008a). A cross-modal system linking primary auditory and visual cortices. Hum. Brain Mapp. 29, 848–857. 10.1002/hbm.20560. 18412133 PMC2605422

[ref29] Eckert M. A. Walczak A. Ahlstrom J. Denslow S. Horwitz A. Dubno J. R. (2008b). Age-related effects on word recognition: reliance on cognitive control systems with structural declines in speech-responsive cortex. JARO 9, 252–259. 10.1007/s10162-008-0113-3 18274825 PMC2504602

[ref30] Elton A. Gao W. (2014). Divergent task-dependent functional connectivity of executive control and salience networks. Cortex 51, 56–66. 10.1016/j.cortex.2013.10.012, PMID: 24315034

[ref31] Erb J. Henry M. J. Eisner F. Obleser J. (2013). The brain dynamics of rapid perceptual adaptation to adverse listening conditions. J. Neurosci. 33, 10688–10697. 10.1523/JNEUROSCI.4596-12.2013, PMID: 23804092 PMC6618499

[ref32] Erb J. Obleser J. (2013). Upregulation of cognitive control networks in older adults’ speech comprehension. Front. Syst. Neurosci. 7:116. 10.3389/fnsys.2013.00116, PMID: 24399939 PMC3871967

[ref33] Fitzhugh M. C. Whitehead P. S. Johnson L. Cai J. M. Baxter L. C. Rogalsky C. (2019). A functional MRI investigation of crossmodal interference in an audiovisual Stroop task. PLoS One 14:e0210736. 10.1371/journal.pone.0210736, PMID: 30645634 PMC6333399

[ref34] Fjell A. M. Westlye L. T. Amlien I. Espeseth T. Reinvang I. Raz N.. (2009). High consistency of regional cortical thinning in aging across multiple samples. Cereb. Cortex 19, 2001–2012. 10.1093/cercor/bhn232, PMID: 19150922 PMC2733683

[ref35] Fox M. D. Snyder A. Z. Vincent J. L. Corbetta M. Van Essen D. C. Raichle M. E. (2005). The human brain is intrinsically organized into dynamic, anticorrelated functional networks. Proc. Natl. Acad. Sci. USA 102, 9673–9678. 10.1073/pnas.0504136102, PMID: 15976020 PMC1157105

[ref36] Geerligs L. Maurits N. M. Renken R. J. Lorist M. M. (2014). Reduced specificity of functional connectivity in the aging brain during task performance. Hum. Brain Mapp. 35, 319–330. 10.1002/hbm.22175, PMID: 22915491 PMC6869200

[ref37] Geerligs L. Renken R. J. Saliasi E. Maurits N. M. Lorist M. M. (2015). A brain-wide study of age-related changes in functional connectivity. Cereb. Cortex 25, 1987–1999. 10.1093/cercor/bhu012, PMID: 24532319

[ref38] Glisky E. (2007). “Changes in cognitive function in human aging” in Brain aging: Models, methods, and mechanisms. ed. Riddle D. R. (Boca Raton, FL: CRC Press/Taylor & Francis), 3–20. 21204355

[ref39] Gopinath B. Schneider J. Hartley D. Teber E. McMahon C. M. Leeder S. R.. (2011). Incidence and predictors of hearing aid use and ownership among older adults with hearing loss. Ann. Epidemiol. 21, 497–506. 10.1016/j.annepidem.2011.03.005, PMID: 21514179

[ref40] Harris K. C. Dubno J. R. Keren N. I. Ahlstrom J. B. Eckert M. A. (2009). Speech recognition in younger and older adults: a dependency on low-level auditory cortex. J. Neurosci. 29, 6078–6087. 10.1523/JNEUROSCI.0412-09.2009, PMID: 19439585 PMC2717741

[ref41] Hawkins K. Bottone F. G. Ozminkowski R. J. Musich S. Bai M. Migliori R. J.. (2012). The prevalence of hearing impairment and its burden on the quality of life among adults with Medicare supplement insurance. Qual. Life Res. 21, 1135–1147. 10.1007/s11136-011-0028-z, PMID: 21979244

[ref42] Hickok G. Poeppel D. (2007). The cortical organization of speech processing. Nat. Rev. Neurosci. 8, 393–402. 10.1038/nrn2113, PMID: 17431404

[ref43] Hiscock M. Kinsbourne M. (2011). Attention and the right-ear advantage: what is the connection? Brain Cogn. 76, 263–275. 10.1016/j.bandc.2011.03.016, PMID: 21507543

[ref44] Hugdahl K. Westerhausen R. (2016). Speech processing asymmetry revealed by dichotic listening and functional brain imaging. Neuropsychologia 93, 466–481. 10.1016/j.neuropsychologia.2015.12.011, PMID: 26706774

[ref45] Husain F. T. Carpenter-Thompson J. R. Schmidt S. A. (2014). The effect of mild-to-moderate hearing loss on auditory and emotion processing networks. Front. Syst. Neurosci. 8:10. 10.3389/fnsys.2014.00010, PMID: 24550791 PMC3912518

[ref46] Husain F. T. Medina R. E. Davis C. W. Szymko-Bennett Y. Simonyan K. Pajor N. M.. (2011). Neuroanatomical changes due to hearing loss and chronic tinnitus: a combined VBM and DTI study. Brain Res. 1369, 74–88. 10.1016/j.brainres.2010.10.095, PMID: 21047501 PMC3018274

[ref47] Jerger J. Chmiel R. Allen J. Wilson A. (1994). Effects of age and gender on dichotic sentence identification. Ear Hear. 15, 274–286. 10.1097/00003446-199408000-00002, PMID: 7958527

[ref48] Jerger J. Chmiel R. Stach B. Spretnjak M. (1993). Gender affects audiometric shape in presbyacusis. J. Am. Acad. Audiol. 4, 42–49. PMID: 8422482

[ref49] Lightfoot G. (2016). Summary of the N1-P2 cortical auditory evoked potential to estimate the auditory threshold in adults. Semin. Hear. 37, 1–8. 10.1055/s-0035-1570334, PMID: 27587918 PMC4910570

[ref50] Lin F. R. Ferrucci L. An Y. Goh J. O. Doshi J. Metter E. J.. (2014). Association of hearing impairment with brain volume changes in older adults. NeuroImage 90, 84–92. 10.1016/j.neuroimage.2013.12.059, PMID: 24412398 PMC3951583

[ref51] Lin F. R. Metter E. J. O’Brien R. J. Resnick S. M. Zonderman A. B. Ferrucci L. (2011a). Hearing loss and incident dementia. Arch. Neurol. 68, 214–220. 10.1001/archneurol.2010.362 21320988 PMC3277836

[ref52] Lin F. R. Thorpe R. Gordon-Salant S. Ferrucci L. (2011b). Hearing loss prevalence and risk factors among older adults in the United States. J. Gerontol. A Biol. Sci. Med. Sci. 66A, 582–590. 10.1093/gerona/glr002 PMC3074958 21357188

[ref53] Loughrey D. G. Kelly M. E. Kelley G. A. Brennan S. Lawlor B. A. (2017). Association of age-related hearing loss with cognitive function, cognitive impairment, and dementia: a systematic review and meta-analysis. JAMA Otolaryngol. Head Neck Surg. 144, 115–126. 10.1001/jamaoto.2017.2513 PMC5824986 29222544

[ref54] Milham M. P. Banich M. T. Webb A. Barad V. Cohen N. J. Wszalek T.. (2001). The relative involvement of anterior cingulate and prefrontal cortex in attentional control depends on nature of conflict. Cogn. Brain Res. 12, 467–473. 10.1016/S0926-6410(01)00076-3, PMID: 11689307

[ref55] Moore D. R. Edmondson-Jones M. Dawes P. Fortnum H. McCormack A. Pierzycki R. H.. (2014). Relation between speech-in-noise threshold, hearing loss and cognition from 40–69 years of age. PLoS One 9:e107720. 10.1371/journal.pone.0107720, PMID: 25229622 PMC4168235

[ref56] Morzaria S. Westerberg B. D. Kozak F. K. (2004). Systematic review of the etiology of bilateral sensorineural hearing loss in children. Int. J. Pediatr. Otorhinolaryngol. 68, 1193–1198. 10.1016/j.ijporl.2004.04.013, PMID: 15302152

[ref57] Murphy K. Birn R. M. Bandettini P. A. (2013). Resting-state fMRI confounds and cleanup. NeuroImage 80, 349–359. 10.1016/j.neuroimage.2013.04.001, PMID: 23571418 PMC3720818

[ref58] Onoda K. Ishihara M. Yamaguchi S. (2012). Decreased functional connectivity by aging is associated with cognitive decline. J. Cogn. Neurosci. 24, 2186–2198. 10.1162/jocn_a_00269, PMID: 22784277

[ref59] Peelle J. E. (2018). Listening effort: how the cognitive consequences of acoustic challenge are reflected in brain and behavior. Ear Hear. 39, 204–214. 10.1097/AUD.0000000000000494, PMID: 28938250 PMC5821557

[ref60] Peelle J. E. Troiani V. Grossman M. Wingfield A. (2011). Hearing loss in older adults affects neural systems supporting speech comprehension. J. Neurosci. 31, 12638–12643. 10.1523/JNEUROSCI.2559-11.2011, PMID: 21880924 PMC3175595

[ref61] Petersen S. E. Posner M. I. (2012). The attention system of the human brain: 20 years after. Annu. Rev. Neurosci. 35, 73–89. 10.1146/annurev-neuro-062111-150525, PMID: 22524787 PMC3413263

[ref62] Profant O. Tintěra J. Balogová Z. Ibrahim I. Jilek M. Syka J. (2015). Functional changes in the human auditory cortex in ageing. PLoS One 10:e0116692. 10.1371/journal.pone.0116692, PMID: 25734519 PMC4348517

[ref63] Puschmann S. Thiel C. M. (2017). Changed crossmodal functional connectivity in older adults with hearing loss. Cortex 86, 109–122. 10.1016/j.cortex.2016.10.014, PMID: 27930898

[ref64] Rauschecker J. P. Scott S. K. (2009). Maps and streams in the auditory cortex: nonhuman primates illuminate human speech processing. Nat. Neurosci. 12, 718–724. 10.1038/nn.2331, PMID: 19471271 PMC2846110

[ref65] Raz N. Rodrigue K. M. (2006). Differential aging of the brain: patterns, cognitive correlates and modifiers. Neurosci. Biobehav. Rev. 30, 730–748. 10.1016/j.neubiorev.2006.07.001, PMID: 16919333 PMC6601348

[ref66] Roberts K. L. Hall D. A. (2008). Examining a supramodal network for conflict processing: a systematic review and novel functional magnetic resonance imaging data for related visual and auditory stroop tasks. J. Cogn. Neurosci. 20, 1063–1078. 10.1162/jocn.2008.20074, PMID: 18211237

[ref67] Rockland K. S. Ojima H. (2003). Multisensory convergence in calcarine visual areas in macaque monkey. Int. J. Psychophysiol. 50, 19–26. 10.1016/S0167-8760(03)00121-1, PMID: 14511833

[ref68] Rosemann S. Thiel C. M. (2019). The effect of age-related hearing loss and listening effort on resting state connectivity. Sci. Rep. 9:2337. 10.1038/s41598-019-38816-z 30787339 PMC6382886

[ref69] Roup C. M. Wiley T. L. Wilson R. H. (2006). Dichotic word recognition in young and older adults. J. Am. Acad. Audiol. 17, 230–240. 10.3766/jaaa.17.4.2, PMID: 16761698

[ref70] Schmidt S. A. Akrofi K. Carpenter-Thompson J. R. Husain F. T. (2013). Default mode, dorsal attention and auditory resting state networks exhibit differential functional connectivity in tinnitus and hearing loss. PLoS One 8:e76488. 10.1371/journal.pone.0076488, PMID: 24098513 PMC3788711

[ref71] Seeley W. W. Menon V. Schatzberg A. F. Keller J. Glover G. H. Kenna H.. (2007). Dissociable intrinsic connectivity networks for salience processing and executive control. J. Neurosci. 27, 2349–2356. 10.1523/JNEUROSCI.5587-06.2007, PMID: 17329432 PMC2680293

[ref72] Smith S. M. Fox P. T. Miller K. L. Glahn D. C. Fox P. M. Mackay C. E. (2009). Correspondence of the brain’s functional architecture during activation and rest. PNAS 106, 13040–13045. 10.1073/pnas.0905267106 19620724 PMC2722273

[ref73] Summerfield Q. (1992). Lipreading and audio-visual speech perception. Philos. Trans. R. Soc. Lond. Ser. B Biol. Sci. 335, 71–78. 10.1098/rstb.1992.0009, PMID: 1348140

[ref74] Tadros S. F. Frisina S. T. Mapes F. Kim S. Frisina D. R. Frisina R. D. (2005). Loss of peripheral right-ear advantage in age-related hearing loss. Audiol. Neurootol. 10, 44–52. 10.1159/000082307, PMID: 15567914

[ref75] Tardif E. Clarke S. (2001). Intrinsic connectivity of human auditory areas: a tracing study with DiI. Eur. J. Neurosci. 13, 1045–1050. 10.1046/j.0953-816x.2001.01456.x, PMID: 11264678

[ref76] Tomasi D. Volkow N. D. (2012a). Aging and functional brain networks. Mol. Psychiatry 17, 549–558. 10.1038/mp.2011.81 PMC3193908 21727896

[ref77] Tomasi D. Volkow N. D. (2012b). Resting functional connectivity of language networks: characterization and reproducibility. Mol. Psychiatry 17, 841–854. 10.1038/mp.2011.177 22212597 PMC3323720

[ref78] Tun P. A. Benichov J. Wingfield A. (2010). Response latencies in auditory sentence comprehension: effects of linguistic versus perceptual challenge. Psychol. Aging 25, 730–735. 10.1037/a0019300, PMID: 20853977 PMC3020665

[ref79] Upadhyay J. Silver A. Knaus T. A. Lindgren K. A. Ducros M. Kim D.-S.. (2008). Effective and structural connectivity in the human auditory cortex. J. Neurosci. 28, 3341–3349. 10.1523/JNEUROSCI.4434-07.2008, PMID: 18367601 PMC6670606

[ref80] Vaden K. I. Kuchinsky S. E. Ahlstrom J. B. Dubno J. R. Eckert M. A. (2015). Cortical activity predicts which older adults recognize speech in noise and when. J. Neurosci. 35, 3929–3937. 10.1523/JNEUROSCI.2908-14.2015, PMID: 25740521 PMC4348188

[ref81] Van Dijk K. R. A. V. Hedden T. Venkataraman A. Evans K. C. Lazar S. W. Buckner R. L. (2010). Intrinsic functional connectivity as a tool for human connectomics: theory, properties, and optimization. J. Neurophysiol. 103, 297–321. 10.1152/jn.00783.2009, PMID: 19889849 PMC2807224

[ref82] Vidal-Piñeiro D. Valls-Pedret C. Fernández-Cabello S. Arenaza-Urquijo E. M. Sala-Llonch R. Solana E.. (2014). Decreased default mode network connectivity correlates with age-associated structural and cognitive changes. Front. Aging Neurosci. 6:256. 10.3389/fnagi.2014.00256, PMID: 25309433 PMC4174767

[ref83] Walhovd K. B. Fjell A. M. Reinvang I. Lundervold A. Dale A. M. Eilertsen D. E.. (2005). Effects of age on volumes of cortex, white matter and subcortical structures. Neurobiol. Aging 26, 1261–1270. 10.1016/j.neurobiolaging.2005.05.020, PMID: 16005549

[ref84] Wechsler D. Coalson D. L. Raiford S. E. (2008). WAIS-IV: Wechsler adult intelligence scale. San Antioni, TX: Pearson.

[ref85] Westerhausen R. Bless J. Kompus K. (2015). Behavioral laterality and aging: the free-recall dichotic-listening right-ear advantage increases with age. Dev. Neuropsychol. 40, 313–327. 10.1080/87565641.2015.1073291, PMID: 26285097

[ref86] Whitfield-Gabrieli S. Nieto-Castanon A. (2012). Conn: a functional connectivity toolbox for correlated and anticorrelated brain networks. Brain Connect. 2, 125–141. 10.1089/brain.2012.0073, PMID: 22642651

[ref87] Wild C. J. Yusuf A. Wilson D. E. Peelle J. E. Davis M. H. Johnsrude I. S. (2012). Effortful listening: the processing of degraded speech depends critically on attention. J. Neurosci. 32, 14010–14021. 10.1523/JNEUROSCI.1528-12.2012, PMID: 23035108 PMC6704770

[ref88] Wilson S. M. Saygin A. P. Sereno M. I. Iacoboni M. (2004). Listening to speech activates motor areas involved in speech production. Nat. Neurosci. 7, 701–702. 10.1038/nn1263, PMID: 15184903

[ref89] Yamasoba T. Lin F. R. Someya S. Kashio A. Sakamoto T. Kondo K. (2013). Current concepts in age-related hearing loss: epidemiology and mechanistic pathways. Hear. Res. 303, 30–38. 10.1016/j.heares.2013.01.021, PMID: 23422312 PMC3723756

